# Resilience to Nested Crises: The Effects of the Beirut Explosion on COVID-19 Safety Protocol Adherence During Humanitarian Assistance to Refugees

**DOI:** 10.3389/fpubh.2022.870158

**Published:** 2022-07-05

**Authors:** Stephanie Nawyn, Ezgi Karaoğlu, Stephen Gasteyer, Rania Mansour, Ali Ghassani, Sandra Marquart-Pyatt

**Affiliations:** ^1^Department of Sociology, College of Social Science, Michigan State University, East Lansing, MI, United States; ^2^Program of Social Work, Doha Institute for Graduate Studies, Doha, Qatar; ^3^Faculty of Health Sciences, Amel Association International, American University of Beirut, Beirut, Lebanon; ^4^Department of Geography College of Social Science, Michigan State University, East Lansing, MI, United States; ^5^Department of Geography, Environment, and Spatial Sciences and Department of Political Science, College of Social Science, Michigan State University, East Lansing, MI, United States

**Keywords:** refugees, Middle East, Turkey, COVID-19, Arab

## Abstract

To provide services safely to refugees during the COVID-19 pandemic, humanitarian non-governmental organizations (NGOs) have instituted public health safety protocols to mitigate the risk of spreading the SARS-CoV-2 virus. However, it can be difficult for people to adhere to protocols under the best of circumstances, and in situations of nested crises, in which one crisis contributes to a cascade of additional crises, adherence can further deteriorate. Such a nested crises situation occurred in Beirut, Lebanon, when a massive explosion in the city injured or killed thousands and destroyed essential infrastructure. Using data from a study on COVID-19 safety protocol adherence during refugee humanitarian assistance in Lebanon, Jordan, and Turkey, we conduct a cross-country comparison to determine whether the nested crises in Beirut led to a deterioration of protocol adherence–the “fragile rationalism” orientation–or whether adherence remained robust–the “collective resilience” orientation. We found greater evidence for collective resilience, and from those findings make public health recommendations for service provision occurring in disaster areas.

## Introduction

To provide services safely to refugees during the COVID-19 pandemic, humanitarian non-governmental organizations (NGOs) have instituted public health safety protocols to mitigate the risk of spreading the SARS-CoV-2 virus. However, those protocols are not always followed, with a number of mitigating conditions presenting barriers to protocol adherence. This is especially true in locations where political instability, conflict, and economic hardships are prevalent. Given that the vast majority of refugees have sought protection in low- and middle-income countries that are often vulnerable to these kinds of circumstances, humanitarian stakeholders need to consider how such mitigating conditions might shape COVID-19 safety protocol adherence.

On the evening of August 4, 2020, a massive explosion in a warehouse along Beirut's port occurred that leveled a good portion of the city and caused major disruptions in a number of city services and destruction of physical infrastructure, including the destruction of three hospitals. Several days after the explosion and following protests over government mismanagement, the Lebanese government leadership stepped down. The explosion and political instability increased currency volatility of the Lebanese pound, exacerbating what was already a crisis situation for Lebanese citizens and the large number of refugees living in the country.

This paper is part of a larger study on COVID-19 protocols during refugee assistance in Turkey, Lebanon, and Jordan, in which we examined the adherence to protocols designed to reduce the risk of SARS-CoV-2 infection (specifically social distancing, mask wearing, and hand hygiene) during the summer of 2020. Because the Beirut explosion happened at the approximate halfway point during the data collection, we were able to analyze protocol adherence before and after the explosion, comparing Beirut to other parts of the Middle East. In this way, we can ascertain the probable effects of the explosion on COVID-19 protocol adherence. This paper addresses the question, is the Beirut blast associated with a change in adherence to COVID-19 prevention protocols by refugees and service providers?

### Health Behaviors and Responses to Crises

Previous work on nested or cascading crises, in which one crisis produces other crises, has demonstrated detrimental effects of health outcomes. For example, Shrecker (1) argued that the 2008 global recession led to increases in food prices and devaluing of local currencies, which contributed to an increase in food insecurity and chronic undernutrition, and in the long-term resulted in a number of deleterious health outcomes. Robinson et al. (2) argues that the impacts of the COVID-19 outbreak themselves constitute a condition of cascading crises of economic insecurity, mental health problems, addiction, and crises of governance, security, and discourse including misinformation. Quigley et al. (3) noted the need for preparations to be made to mitigate the impacts of natural disasters (floods, hurricanes, cyclones, earthquakes, etc.) that occurred on top of COVID-19. But less research has investigated how cascading crises impact health *behaviors*. Marck et al. (4) found that Australians diagnosed with multiple sclerosis experienced deterioration in a number of health behaviors during the summer bushfires in 2019–2020 and the COVID-19 pandemic, including reduced physical activity, increased alcohol consumption, and disrupted sleep patterns. Bell et al. (5) linked existing health survey data with federal disaster declarations at the county-level data and found an association among older adults between exposure to a disaster and reduced physical activity but no association with smoking. These studies were limited in that they used a cross-sectional sample, and thus were not able to measure changes over time. Using longitudinal data, Ásgeirsdóttir et al. (6) found some decline in health behaviors but generally increases in positive health behaviors following the 2008 global recession.

In assessing how people respond to crises such as a pandemic, Reicher and Bauld (7) describe two competing psychological orientations; fragile rationalism and collective resilience. Fragile rationalism is denoted by people's inability to accurately understand risk, and under duress individuals are less likely to follow sound public health practices. In a fragile rationalist frame, inaccurately perceiving threats is a key behavioral problem. Writing in the wake of the influenza epidemic in the early twentieth century, Soper (8) argued that pandemics were difficult to contain because of poor risk perception, the biological disinclination to self isolate, and the unconscious tendency of people to act in ways that endanger themselves and others. Van Bavel et al. (9), using Soper (8) as a reference point, provided a detailed literature review of the scholarship on practicing risk avoidance protocols in the ensuing 100 years. Their analysis identified “threat perception, social context, science communication, aligning individual and collective interests, leadership, and stress and coping as the areas of research focus” as areas of particular importance in understanding reaction to the COVID-19 pandemic (p. 467).

Threat perception is driven by fear, and human fear of threats has evolved as a mechanism to ensure survival (10). Threat perception is associated with how people assess multiple perceived risks—they will act based on what they fear most. A large body of the literature argues that “risk perception is a subjective psychological construct that is influenced by cognitive, emotional, social, cultural, and individual variation both between individuals and between different countries” [(11), p. 995]. Slovic (12) argues that people perceive things over which they do not have control to be more risky. Refugees and staff serving refugees in Lebanon were already in a position of assessing multiple risks to survival before the arrival of the COVID-19, and that these multiple risks of food, water, and economic security had only been exacerbated over time (13, 14).

The alternative psychological orientation is collective resilience, in which a population exhibits collective resilience in the face of multiple crises, with positive health behaviors maintaining or improving over time despite crisis conditions. Elcheroth and Drury (15) review the literature on responses to pandemics and identify conditions under which populations may exhibit collective resilience. Included among those conditions were a perception of a common identity with the persons issuing public health protocols, the preservation of ordinary social roles and relationships, and the preservation of social ties during the pandemic. Williams and John (16) delineate between personal and collective resilience, describing the latter as how groups of people “expect solidarity and cohesion, and thereby coordinate and draw upon collective sources of practical and emotional support adaptively to deal with an emergency or disaster” (p. 294).

Personal and collective resilience to disasters are both strongly influenced by institutional resilience and disaster preparedness (17, 18). Institutional preparedness involves having in place the protocols and practices that facilitate continued delivery of services in the context of compounding disasters (17–19). Such protocols can improve response to disasters and continued service delivery, even in the context of overcrowding. However, when protocols for continued delivery of service were not in place, conditions of overcrowding could exacerbate chaotic breakdowns of adherence to health protocols (20).

Together, this research indicates that maintaining important social institutions and relationships, fostering a sense of collective fate, and receiving public health guidance from people one identifies with can increase the likelihood that a population will effectively manage crisis conditions.

In this paper, we address the competing orientations summarized above. Frame #1, “fragile rationalism” is that during cascading crises there will be a degraded adherence to public health safety protocols. The underlying assumption is that the cascading crises will lead to people losing trust in the authorities that are recommending or mandating safety protocols, and people will simply abandon those protocols in face of that degraded trust. Frame #2, “collective resilience,” is that in the face of cascading crises people will adopt collective resilience behaviors as a way of coping with the crises and act as a collective with a shared fate, leading to maintained or improved adherence to safety protocols. There is indeed evidence that the chaotic social context in Lebanon led to the Lebanese people losing trust in the central government prior to the pandemic, and prior to the explosion (21, 22). Given this, the question is whether prior political mobilization, and the necessity of reliance on local social networks (21) led to anomic behavior (23) thus supporting the “fragile rationalism” frame, or “collective resilience” through greater collaboration and adherence to safety protocols [(24), p. 66].

We test these different orientations for understanding the effects of nested crises by comparing COVID-19 protocol adherence during humanitarian service provision in Beirut, a location experiencing nested crises during the summer of 2020, to similar service provision in Turkey and Jordan that was not directly impacted by the port explosion in Beirut and the severe economic instability in Lebanon broadly.

### Regional Context

Lebanon, Turkey, and Jordan are among the top refugee hosting countries in the world, together hosting 7.9 million refugees^1^ These three countries host large populations of Syrian and Palestinian refugees, as well as smaller populations of refugees from Iraq, Afghanistan, Iran, and very small populations from other countries. In Turkey, most refugees are Syrian and live in urban settings, outside of camps. In Lebanon and Jordan, Palestinians make up large proportions of the refugee populations, along with Syrians, and many live in long-settled camps that function much like urban settings.

All three countries are facing economic stressors and political instability. And all three countries have dealt with substantial COVID-19 outbreaks, albeit at different times. Turkey experienced the earliest wave, starting in March 2020 and continued increases in identified cases over time. Infections in Lebanon started later, around the first week of July and increased sharply through the beginning of 2021. Fares et al. (14) analyzed data from the Lebanese Ministry of Public Health gathered in late July through mid-August and found that prior to the explosion the positivity rate for COVID-19 tests rose sharply from 2.7 per 100 to 6.4 per 100 afterwards. The hospitalization rate also rose from 139 patients prior to the explosion to 266 patients after the explosion, with an increase in patients admitted to the ICU ward from 36 before the explosion to 75 ICU patients afterwards. In Jordan, the daily numbers of new infections were stable and relatively low from March 2020 through most of the summer. Infections increased slowly starting in mid-August through early September. After the first week of September new infections increased sharply, and Jordan has since experienced two waves of infection. In all three countries, mitigation strategies were implemented that included stay-at-home orders, curfews, and other forms of limits on people's interactions with others in public.

Personal Protection Equipment (PPE) has been available in all three countries, and commonly used by humanitarian NGOs while serving refugees. Due to both devaluation of the Lebanese pound and increased demand, the cost of PPE increased dramatically during the summer of 2020. This included face masks, making it more difficult for refugees in Lebanon to afford masks and leading to the inability of many NGO service providers to make masks available to refugee clients who arrived without them.

The pandemic arrived on the heels of close to a decade of refugee flows not matched since the first part of the Twentieth Century (25). The humanitarian crisis was significantly dramatic in the Middle East region—caused largely by the chaos of the Syrian civil war and ongoing unrest in Iraq. More than 1 million refugees have fled the Syrian war into Lebanon since 2011, making it the country with the world's largest refugee population per capita (26). Given the reticence of the Lebanese authorities to provide designated camps for Syrian refugees, most live in Informal Tented Settlements (ITS) outside Beirut and in the rural areas. Those Syrian refugees who live in Beirut and other large cities live in rented houses.

The explosion of stockpiled explosives at the Port of Beirut, Lebanon was preceded by years of dysfunctional, corrupt, chaotic governance that even before the pandemic of 2020 had become increasingly untenable (25). Political gridlock and corruption has led to multiple crises in sanitation management, specifically garbage disposal, shutoffs and disruptions in basic services such as water and electricity, as well as increasing economic inequality and a central bank crisis. These multiple crises were exacerbated by the influx of refugees from the Syrian civil war. The compounded crises have significantly impacted Lebanese quality of life and wellbeing, leading to sophisticated community organizing calling for an end to corruption and improved governance, including a human chain that stretched across Lebanon in 2019 (21, 22). While all three countries have faced challenges from both the Syrian refugee crisis and the COVID-19 pandemic, Lebanon's political and economic difficulties have been more intense that what has been felt in Turkey or Jordan^2^.

## Materials and Methods

This study uses data collected^3^ from humanitarian assistance organizations serving refugees in Lebanon (Amel Association and National Institution of Social Care and Vocational Training), Turkey (Safa for Development), and Jordan (Altkafal Charity Association), each with multiple service centers. The location of service centers in Turkey were in Konya (central Turkey) and Reyhanli (southeastern Turkey). The service centers in Jordan were three locations in the governorate of Irbid (northeastern Jordan). The service centers in Lebanon were dispersed throughout the country, with four locations in Beirut. The research team includes university researchers in the United States collaborating with NGO coordinators in Lebanon, Turkey, and Jordan; the NGO coordinators in Lebanon and Jordan also held university faculty positions. This team collaborated on the research design and data analysis, allowing for more complete interpretation of the data because a portion of our research team experienced the nested crises first-hand (27).

Data were collected from interviews with NGO staff on how well-refugees and staff practiced social distancing (keeping 2 m distance between each other), wore face masks, and washed or sanitized hands and surfaces during services provided to refugees. Fifteen data collectors conducted interviews, asking a series of closed-ended and open-ended questions to staff either in person, over the phone, or in a few cases through video conferencing. The data collectors asked questions about how frequently safety protocols were followed based on a Likert-type scale; All of the time, Most of the time, Some of the time, or Very little of the time. The questions referred to the services that the staff provided either earlier that day or the previous day (depending upon what time the interview was conducted). Staff were asked to reflect on how frequently refugees maintained social distance and wore masks, how frequently staff followed these protocols around refugees and around other staff, and then how frequently the interviewee personally followed these protocols, all during the specific service referenced in the interview. Staff were also asked how often refugees washed their hands before, during, and after that service, how often refugees used hand sanitizer before, during, and after that service. Staff were also asked about those same hand hygiene protocols for other staff.

In addition to the three primary safety protocol behaviors, we collected data on the location where services were provided, the day of the interview, the type of service, and the refugee populations served. Data collectors entered the interview responses into a Qualtrics database, so that data monitoring could occur in real time throughout the data collection period given the geographic expanse of the research team and study populations.

Using interviews as the observational unit, we constructed comparisons that allowed us to identify similarities and differences in protocol adherence along axes of time, resources, and regions. The findings described in this paper are based on interviews conducted about services in Beirut (319 interviews), comparing those to interviews about services in Turkey (464 interviews) and Jordan (209 interviews). Among 319 interviews conducted in Beirut, 127 (40.8%) of them were the first interview with this certain staff, while 192 (59.2%) of them were at least the second interview conducted with the same staff. In Turkey, among 464 interviews, 423 (91.2%) of them were the first interviews with the staff while the remaining 41 (8.8%) were at least the second time when the same staff was interviewed. For Jordan, among 209, 127 (58.3%) of them were the interviews where the staff was interviewed for the first time, while 83 (41.7%) interviews were at least the second time with the same staff. Interviews were conducted between July 6 and September 15, 2020. Using the date of the interview, we constructed a variable indicating the number of weeks from the start of data collection. Because there were a small number of interviews during the first 2 weeks of data collection, we start week 1 on July 20 and include the next 8 weeks. Measuring time in this way rather than measuring time as a binary (before vs. after the explosion) allows us to observe trends that predated the explosion, so as to avoid attributing changes over time to the explosion that actually started well beforehand. For each protocol, we assigned a numeric value to the four categories of protocol adherence: All of the time = 4, Most of the time = 3, Some of the time = 2, and Very little of the time = 1. For each week, we calculated the average in responses to the frequency of protocol adherence, for a value ranging from 1 to 4, with a higher number indicating better adherence. We examined resource availability comparing Beirut to Turkey and Jordan, and protocol adherence region-wide and comparing Beirut to Turkey and Jordan. We excluded parts of Lebanon outside of Beirut, which may have experienced some ancillary effects of the explosion but would not have been as acutely affected. With a focused comparison of Beirut to Jordan and Turkey, we avoid de-emphasizing the effects of the explosion that may have also impacted other parts of Lebanon but to a lesser extent.

## Results

### Availability of Hand Hygiene Resources

**Figure 1:**
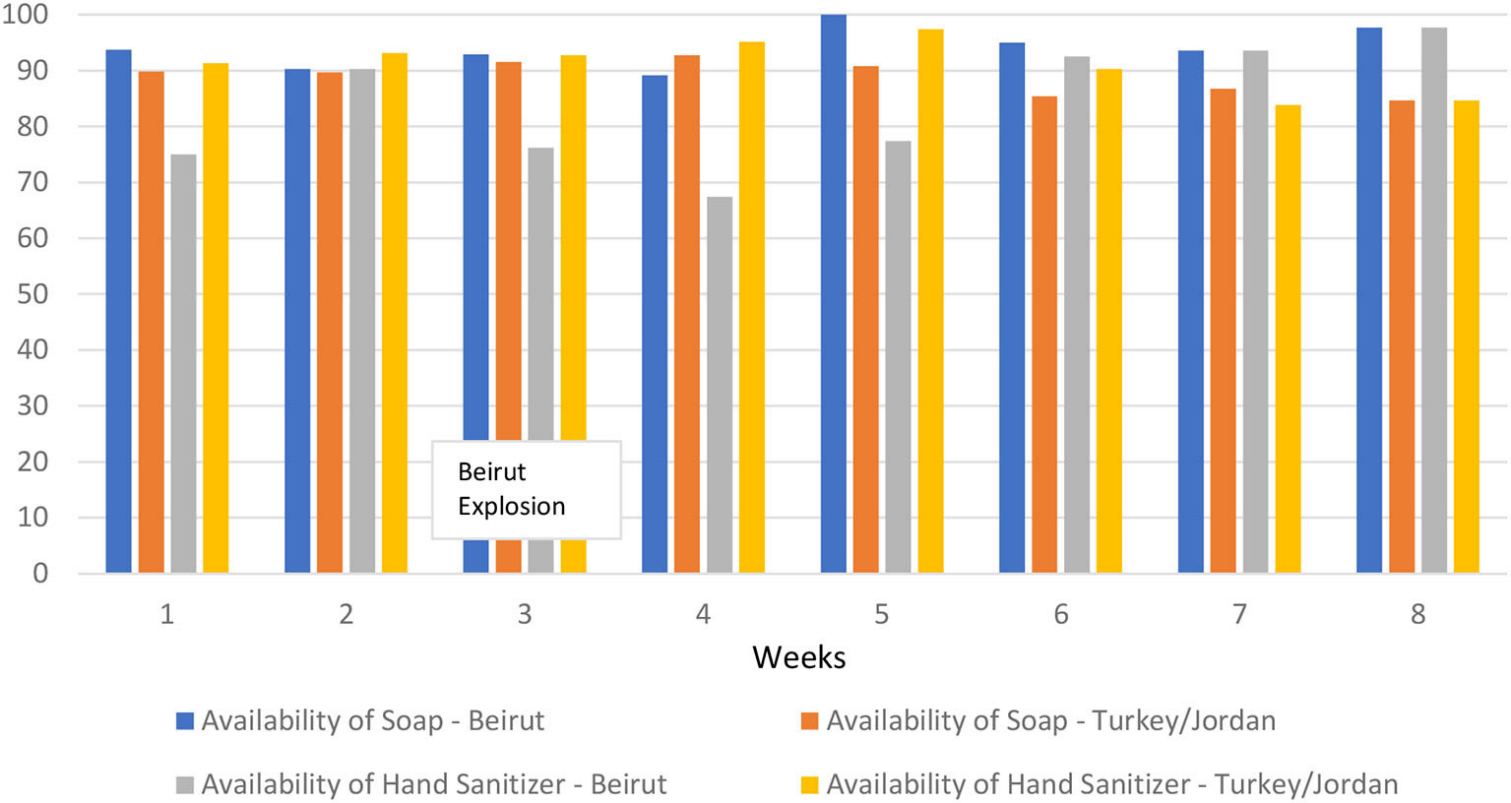
Availability of soap and hand sanitizer by week and location.

Soap, water, and hand sanitizer were broadly available during service provision across the region both before and after the explosion. Figure 1 describes the availability of soap and hand sanitizer during each week of data collection (given similar availability as soap, results for water are not shown but are available upon request). For nearly every week, soap was more commonly available in Beirut than in Turkey and Jordan. The explosion occurred during week 3, yet there was little change in soap availability between week 3 and 4 in Beirut, and during week 5 both were more commonly available in Beirut. Hand sanitizer was less commonly available in Beirut compared to Turkey and Jordan, and availability decreased during weeks 3 and 4. However, availability increased during week 5, and by the final week hand sanitizer was more commonly available in Beirut than in Turkey and Jordan. So while it appears that hand sanitizer availability in Beirut decreased immediately after the explosion, that effect was short-lived.

### Adherence to COVID-19 Safety Protocols

**Figure 2:**
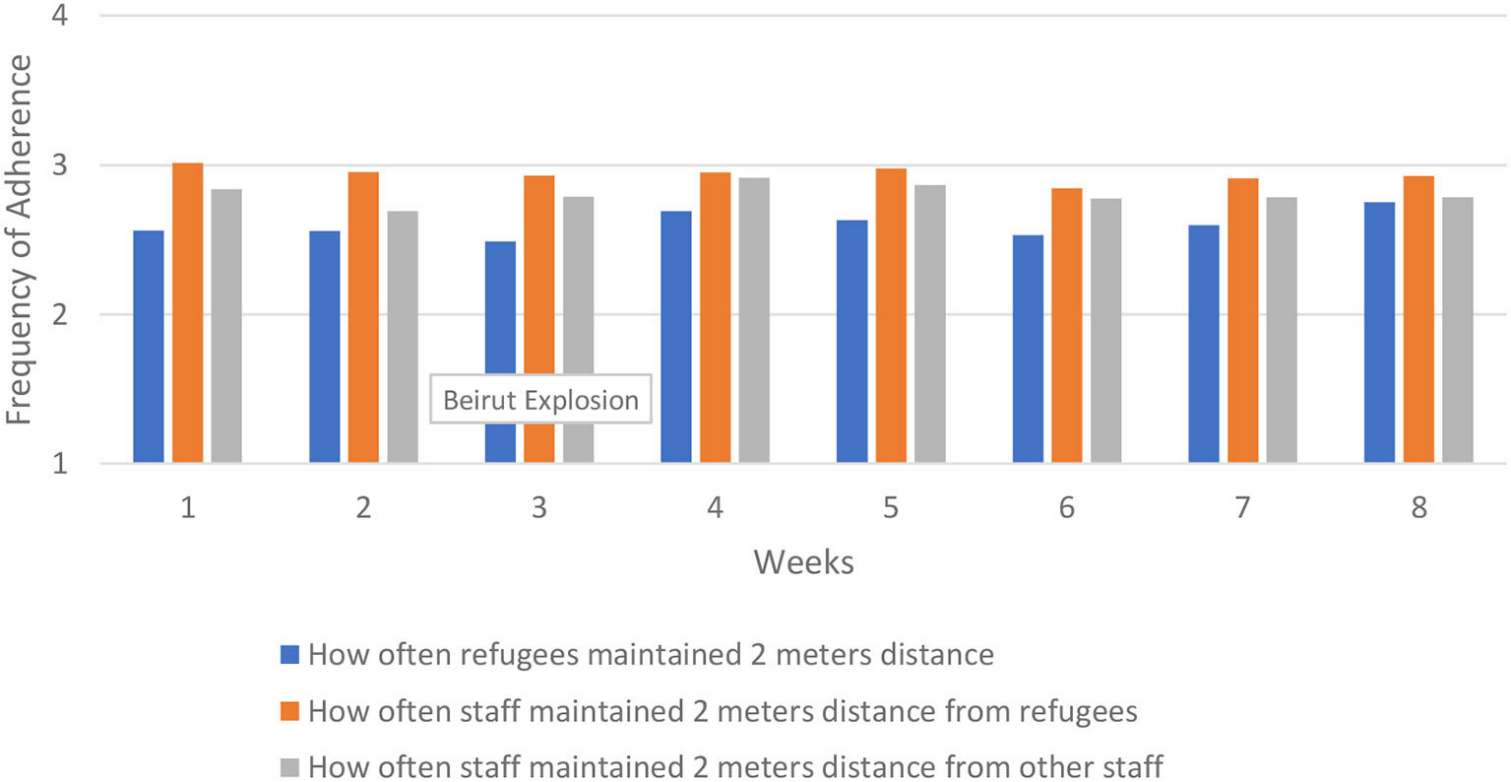
Social distancing across region.

**Figure 3:**
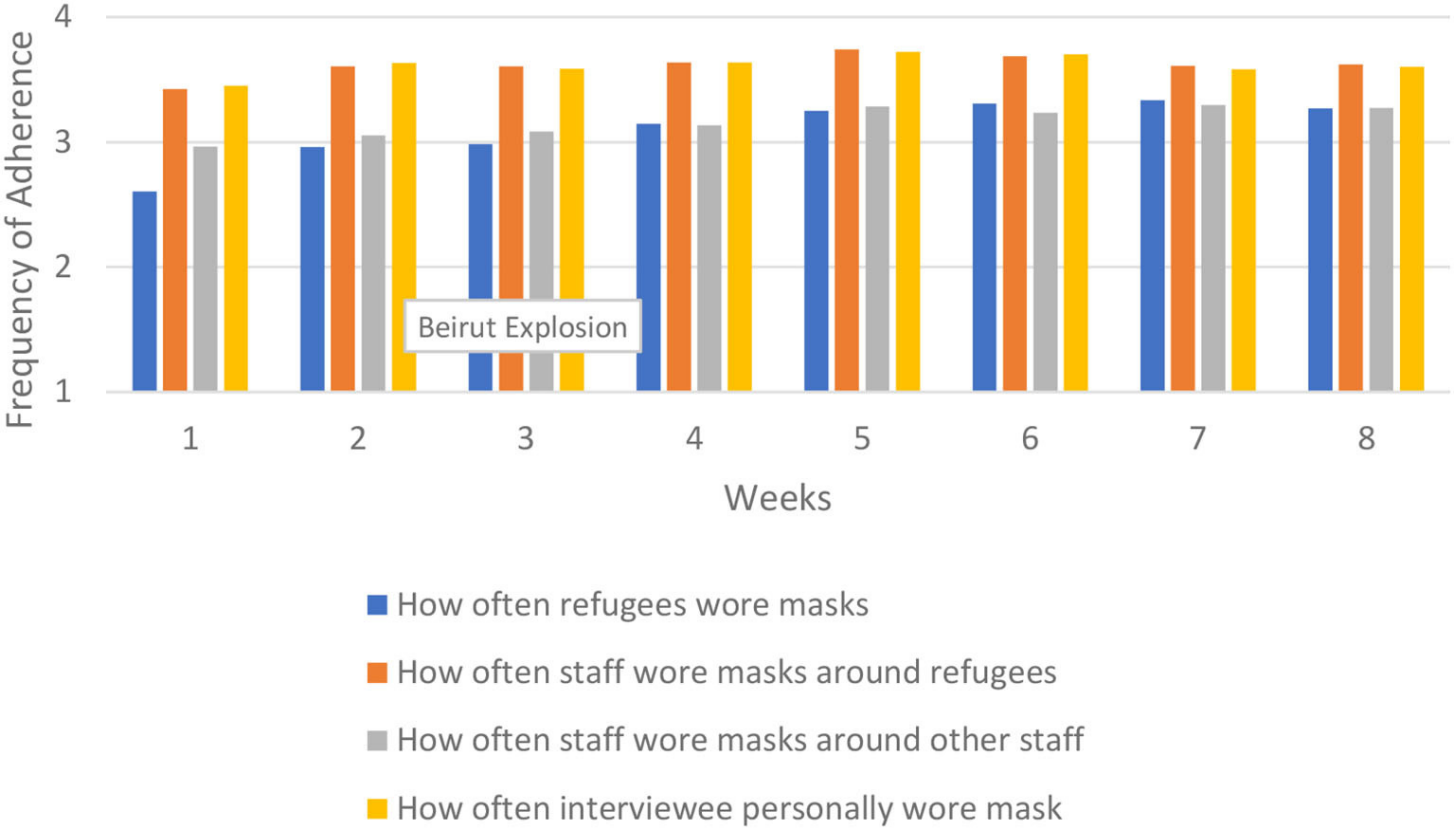
Mask wearing across region.

**Figure 4:**
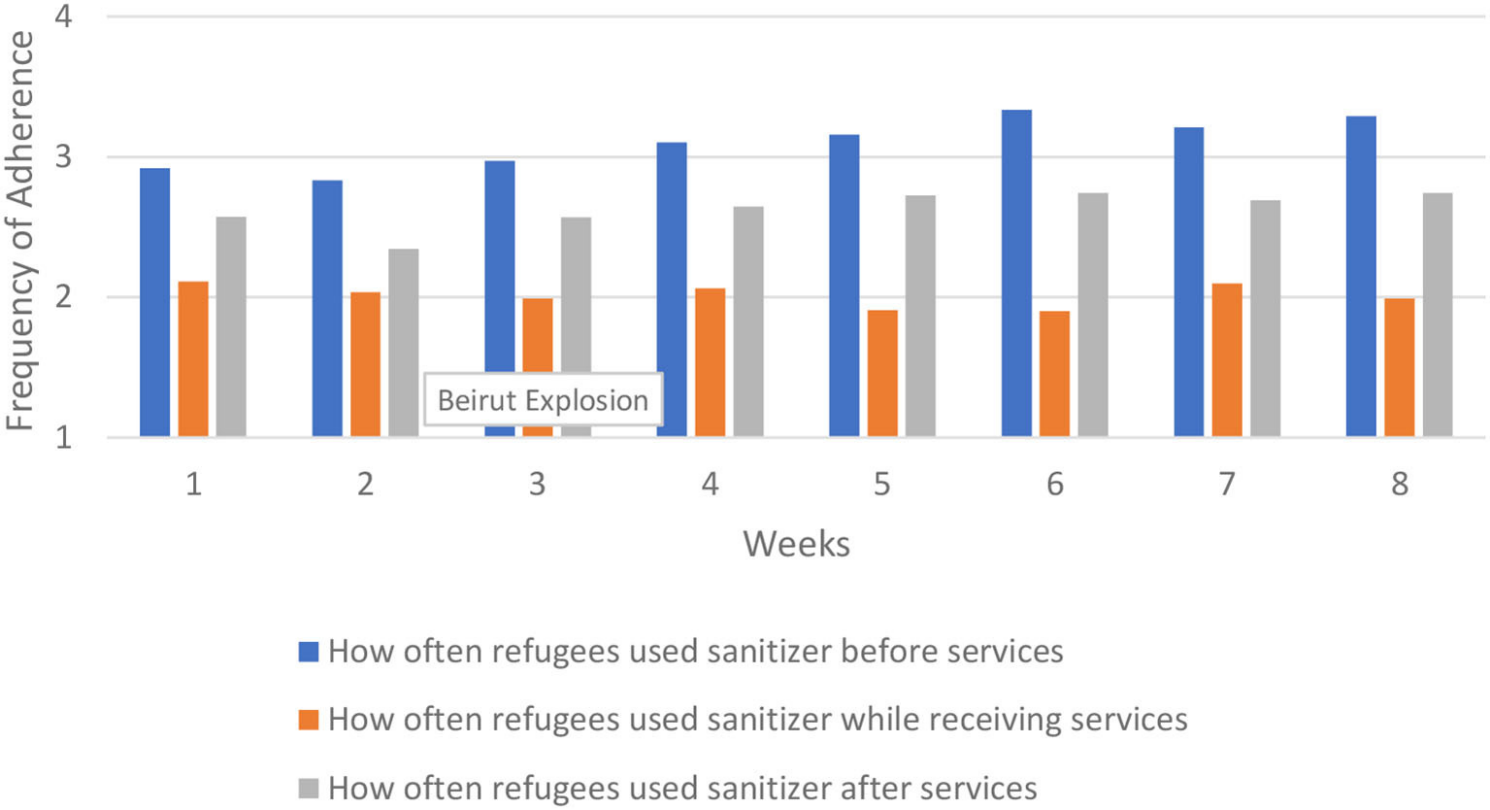
Refugee sanitizer use across region.

**Figure 5:**
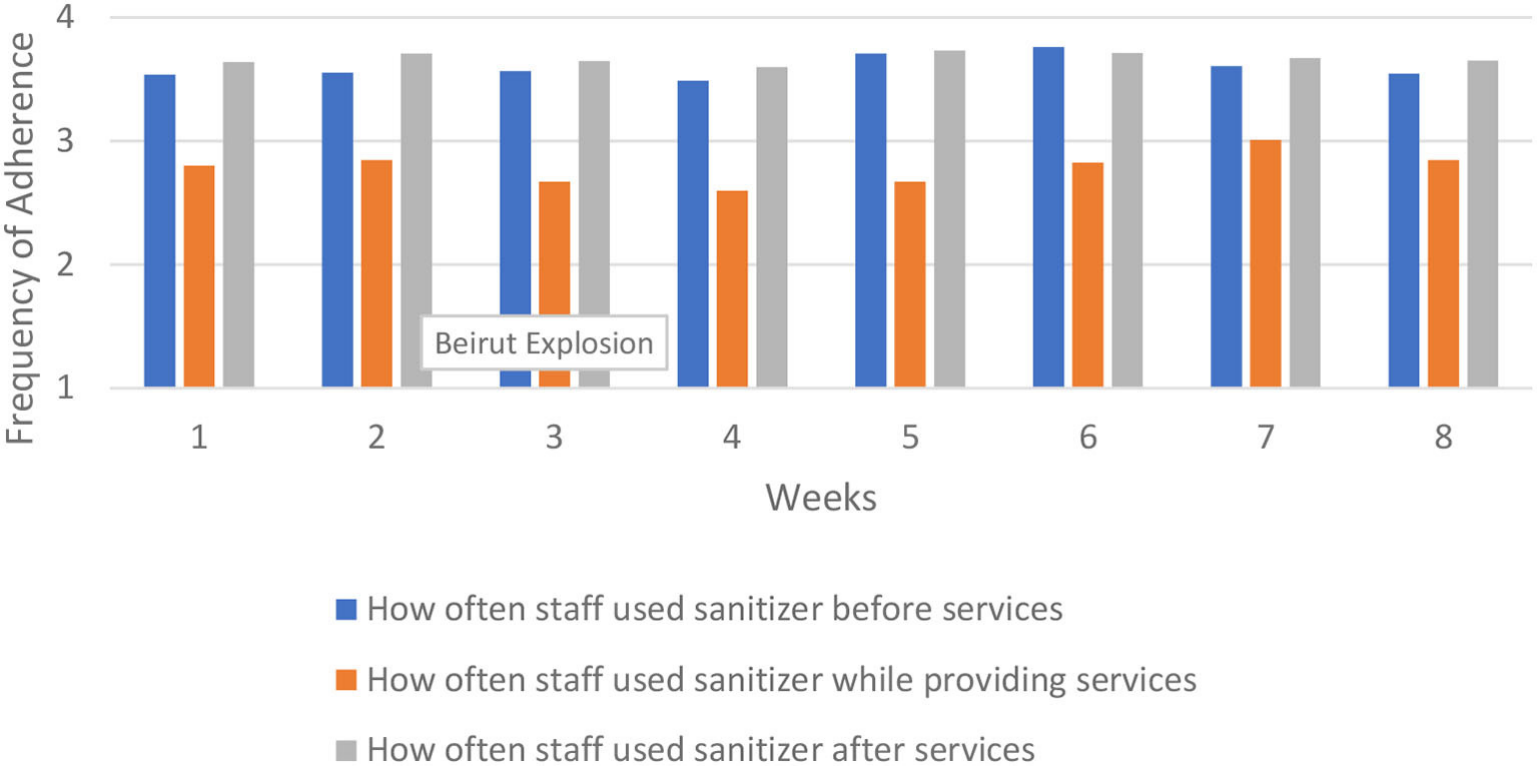
Staff sanitizer use across region.

We first analyze protocol adherence over time across the entire region. This provides an overall picture in patterns of adherence. The results are presented in Figures 2–5. There was some variability in how well different protocols were followed across the region, with a general pattern of staff more frequently following protocols compared to refugees, along with more consistent adherence across time for staff and slight improvement in adherence over time for refugees, particularly in mask wearing and using hand sanitizer before and after services. Social distancing remained generally consistent over time across the region. There was very little change from week to week in how well staff adhered to social distancing protocols, while refugee adherence fluctuated some but improved overall between the first and last week.

Mask wearing improved markedly among refugees, going from an average of 2.60 during the first week to 3.70 during the last week. Staff mask wearing remained high throughout the period but did increase over time in staff wearing masks around other staff.

Our measures of hand washing and sanitizing included how frequently refugees and staff followed these protocols before, during, and after services. Hand sanitizer use and hand washing showed similar patterns for refugees and staff, so we show only the results of the former. Refugees were more likely to use hand sanitizer before services, and least likely during services. Staff were more likely to use hand sanitizer after services, and least likely to use it during services.

Refugees much more frequently used hand sanitizer than washed their hands, particularly before the start of services. The average for refugees using hand sanitizer before services started at 2.92 and increased to 3.29 by the final week (down slightly from 3.33 during week six).

Staff were better at using hand sanitizer compared to refugees, but changed very little over time. Staff's use of hand sanitizer before and after services was consistent throughout the data collection period. While there was some fluctuation in hand sanitizer use during services, adherence during the last week was nearly the same as the first week.

### Comparing Adherence in Beirut vs. Turkey and Jordan

To analyze the effects of the explosion in Beirut, we compare adherence over time in Beirut compared to Turkey and Jordan. The explosion occurred at the beginning of week 3 (which included August 3–9). Thus, if the explosion affected protocol adherence, we would expect to see that effect starting in week 3 or week 4.

**Figure 6:**
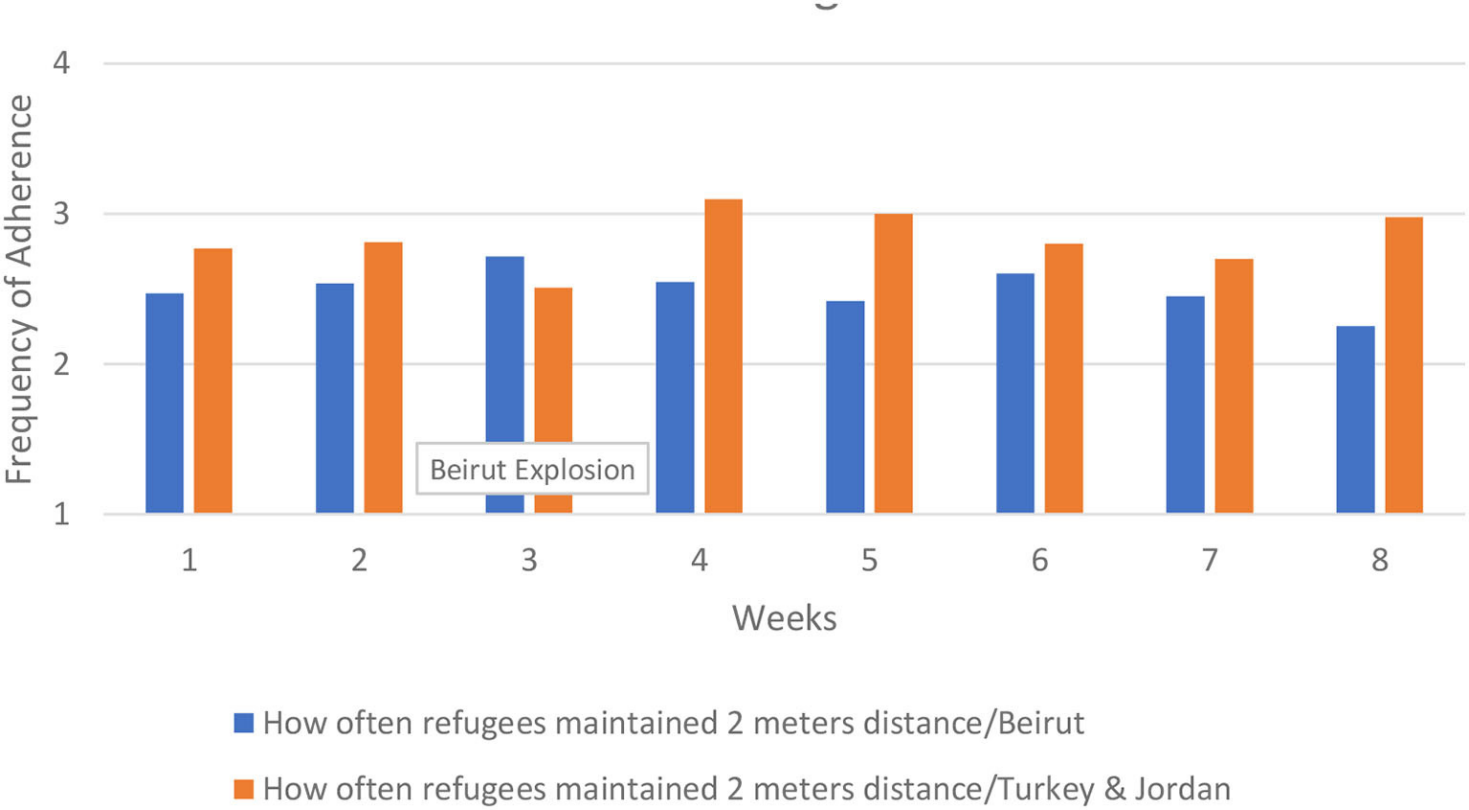
How often refugees maintained social distancing.

**Figure 7:**
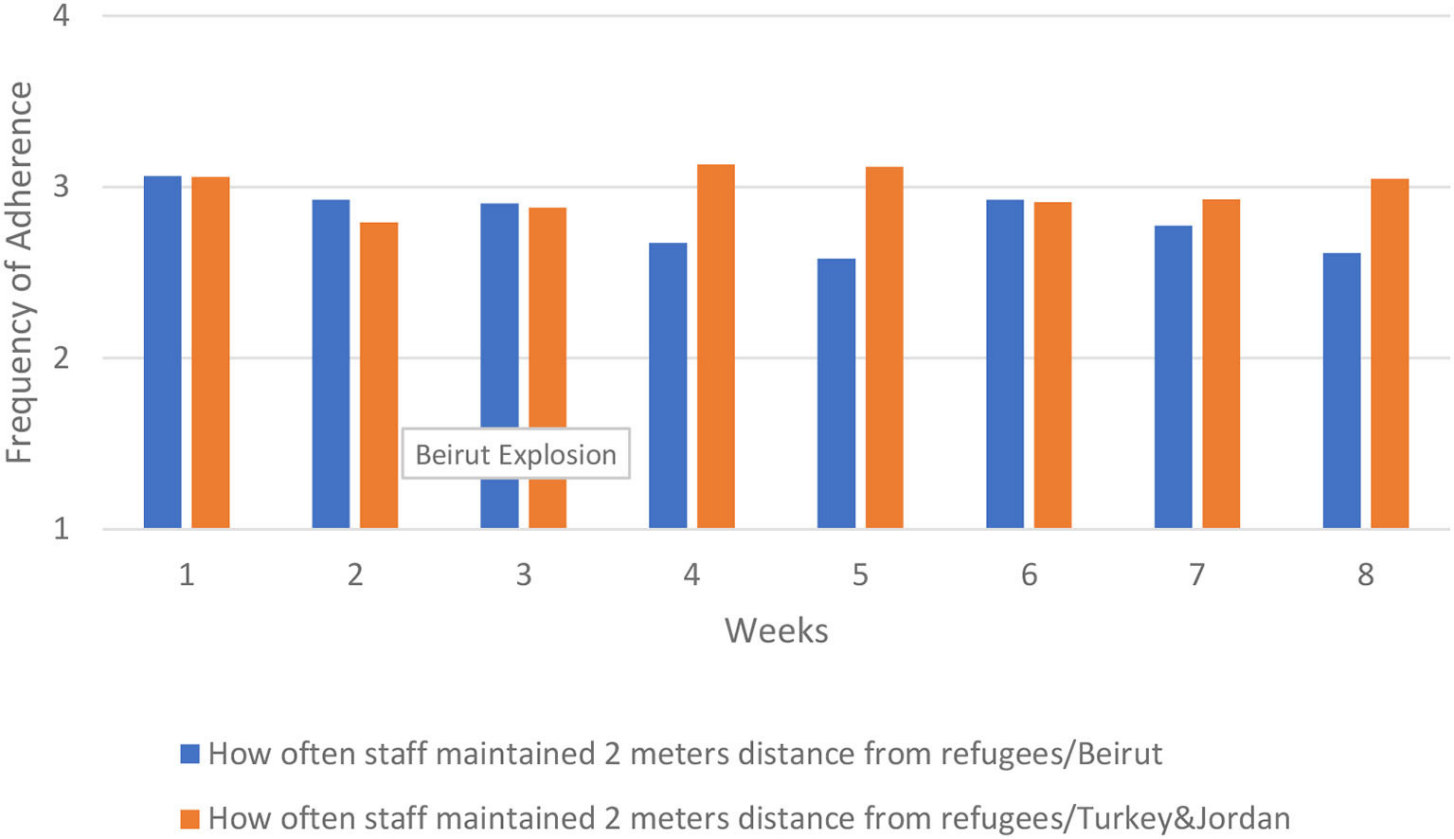
How often staff maintained social distancing from refugees.

**Figure 8:**
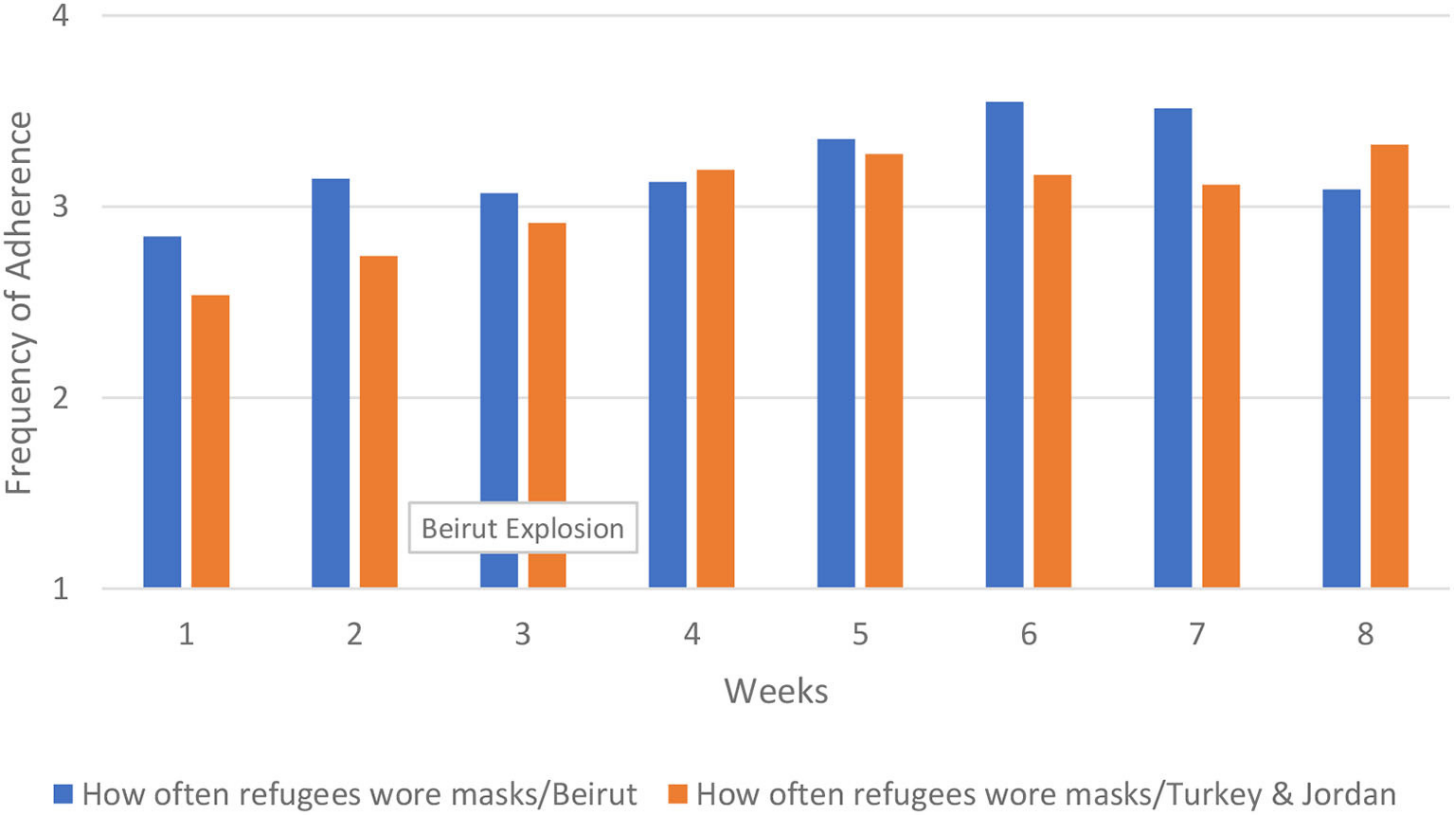
How often refugees wore masks.

**Figure 9:**
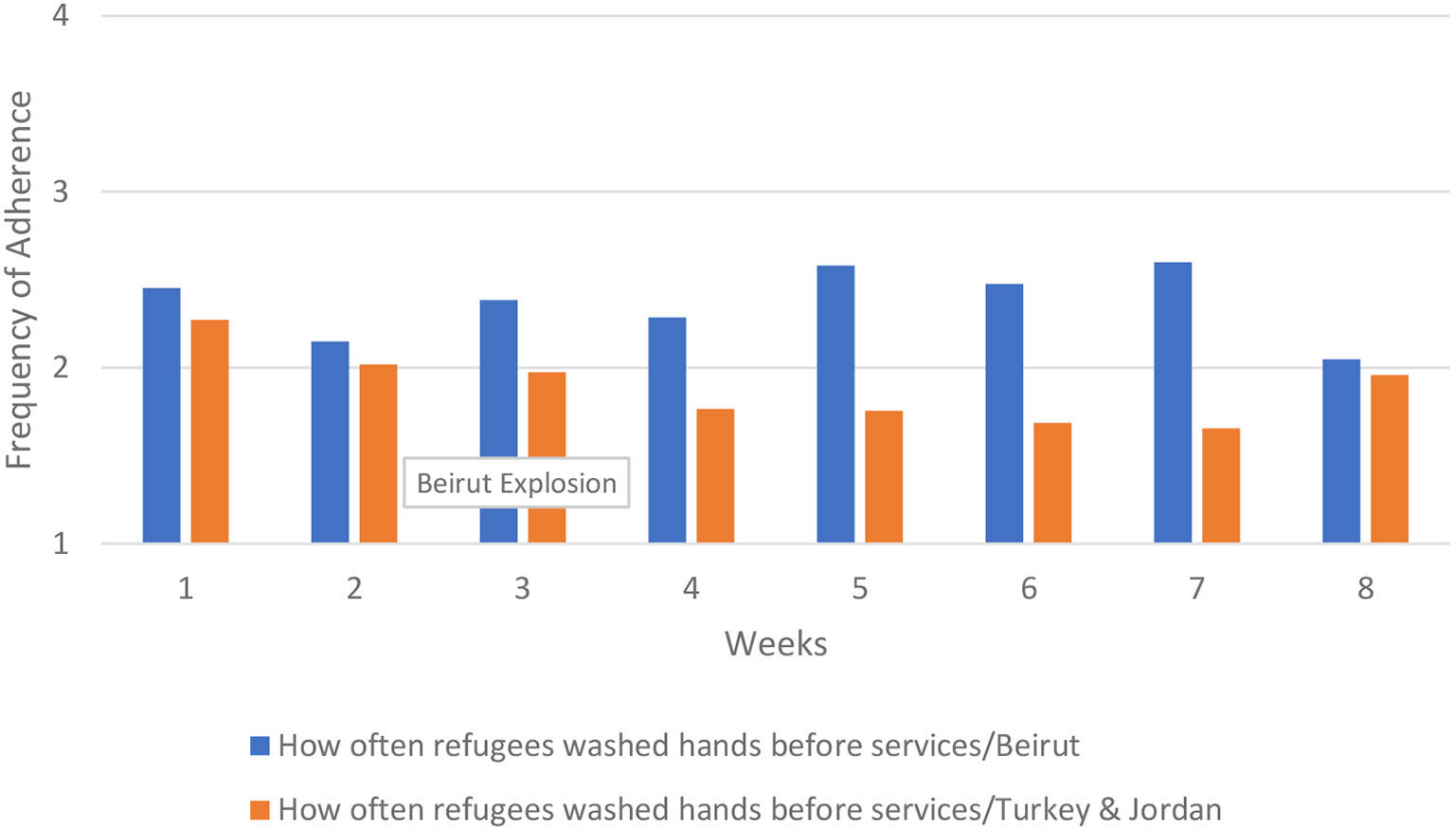
How often refugees washed hands before services.

**Figure 10:**
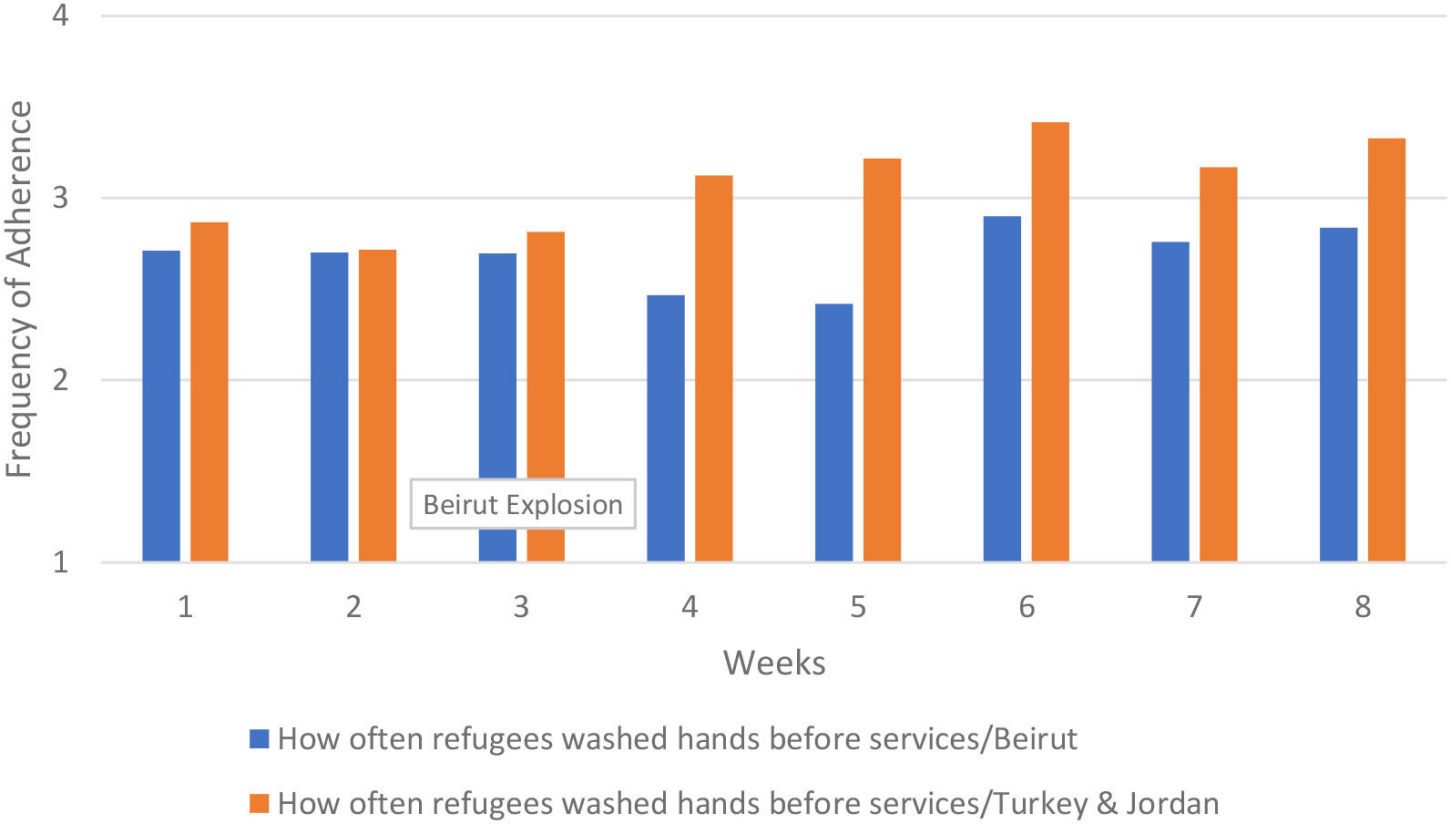
How often refugees used hand sanitizer before services.

Because of the large number of comparisons in these analyses, we limited our presented findings to a select number of figures (all analyses are available from the authors upon request). We present those analyses in Figures 6–**10**. We found four general patterns in adherence to protocols across the 8 weeks of data collection; (1) very little change occurring over time, (2) variable change over time in which adherence was worse in Beirut than in Turkey and Jordan, (3) consistent improvement over time in which adherence was better in Beirut than in Turkey and Jordan, and (4) a notable decrease in adherence in Beirut that corresponded to the time of the explosion, with adherence rebounded by the final week of data collection. We present figures that are emblematic of the second, third, and fourth patterns.

Refugees and staff adherence to social distancing demonstrates a pattern of uneven adherence over time, and ending with adherence in Beirut being worse than in Turkey and Jordan. For example, with social distancing refugee adherence in Beirut fluctuated slightly, ranging from 2.60 in week 6 to 2.25 in week 8, ending with worse adherence compared to Turkey and Jordan. The decline started in week 5, not in week 3 when the explosion occurred. These findings are presented in Figure 6.

Staff adherence to social distancing from refugees decreased after the first week in both Beirut and Turkey/Jordan. In Beirut, adherence continued to decline until week 6, and declined again in week 8. In Turkey and Jordan, there was a general increase in staff's adherence to social distancing from refugees in week 4, ending with better adherence in the final week compared to Beirut. These results are presented in Figure 7.

For mask wearing, adherence in Beirut was better overall compared to Turkey and Jordan, and increased over time. This was particularly true for staff wearing masks around refugees up until the final week when adherence in Beirut and Turkey/Jordan converged. The increase began prior to the explosion and does not seem related to the event. There was a decrease in adherence to all mask wearing protocols in the final week in Beirut, but no change around the time of the explosion in week 3. In Turkey and Jordan, mask wearing by refugees increased sharply from the first to last week, increased somewhat over time for staff wearing masks around other staff, and remained mostly flat for staff wearing masks around refugees. Even with the increases in mask wearing protocol adherence over time, Turkey and Jordan still had less consistent adherence compared to Beirut. These results are shown in Figure 8. The results for staff wearing masks around other staff were similar (results not shown).

For hand hygiene, we examined changes over time in refugees' and staff's washing hands and using hand sanitizer before and after services. Refugees' and staff's adherence to washing hands prior to services was higher in Beirut and improved over time. The improvement occurred largely after the explosion, between week 5 and 6. A similar pattern emerged in hand washing after services. By contrast, in Turkey and Jordan refugees washing their hands before services decreased over time, and refugees washing their hands after services remained flat. For staff, washing hands before and after services changed very little over time in Turkey and Jordan. In Figure 9 we show the results for refugees' adherence to hand washing before services.

In hand sanitizer use we saw the first clear evidence of a decrease in protocol adherence in Beirut that corresponded with the port explosion. There was little change over time in refugees' use of hand sanitizer before services, but in using sanitizer after services there was a marked dip in refugees' adherence in Beirut between weeks 3 and 4. A similar pattern was apparent for staff in hand sanitizer use both before and after services, although the dip was much smaller for staff. By contrast, in Turkey and Jordan hand sanitizer protocol adherence either changed little between weeks 3 and 4, or it improved. However, in all cases the protocol adherence in Beirut rebounded back to pre-explosion levels, although refugees' adherence to those levels remained below those in Turkey and Jordan. The most dramatic changes were seen in refugees' use of hand sanitizer before services, which we show in Figure 10.

## Discussion

While one might expect the massive explosion in Beirut to have a broad detrimental impact on refugee service provision in the city, we found little evidence that the challenges service providers undoubtedly faced had any discernible effect on the hand hygiene resources available nor detracted from the ability of refugees and staff to adhere to COVID-19 safety protocols. On the contrary, protocol adherence during services in Beirut was sometimes better than in our sampled service locations in Turkey and Jordan, and decreases in protocol adherence that occurred did not always directly correspond to the week when the explosion happened. The only instance in which we saw a decrease in protocol adherence in Beirut that corresponded to the explosion was in the use of hand sanitizer, and even in that case protocol adherence improved to near or better than pre-explosion levels by the end of our study period.

Across the region there were general improvements in protocol adherence, particularly among refugees, suggesting that over time people in these service centers become better acclimated to following safety protocols. Our data show that even in the refugee communities in Beirut, there was a similar pattern of acclimation. Our data indicate that the explosion, despite clearly disrupting many aspects of economic and social life, did not lead to a major sustained disruption to improved protocol. Contrary to what would be predicted by the frame of fragile rationalism and much of the literature on health behaviors during nested crises, we did not see a decline in protocol adherence that corresponded to the timeline of the Beirut explosion. Instead, we see evidence of a collective resilience of Beirut's residents that led to steady improvement in adherence to some protocols even with the explosion. Thus, we found support for the collective resilience orientation that Drury et al. (24) described.

When reflecting on the events, our project team members in Lebanon noted the resilience that people in Lebanon have had to develop as a result of multiple, nested crises. The COVID-19 pandemic is experienced by the Lebanese people as a disaster nested within political and economic crises. The explosion, while it led to devastating loss of life and destruction of infrastructure, was just one more crisis nested within the challenges wrought by pandemic, political instability, and economic volatility. Because of this, the explosion did not create the shock effect that it might have if it had occurred in a location that was not already facing multiple challenges.

Our project team's own resilience supports this orientation. While our team in the United States expected data collection in Beirut to slow down following the explosion, our data collectors in Beirut continued to collect data and conducted more interviews in the 2 days following the explosion than the 2 days prior. This occurred even though one of our supervising NGO coordinators in Beirut was attending to damage incurred to her home because of the explosion. While there were new challenges presented by the explosion, our empirical findings and project team experience supports an interpretation that withstanding multiple nested crises builds resilience among people and institutions that protects against “shock” events like that of the Beirut explosion.

While our findings are compelling, there are limitations to this study. These data come from interviews with NGO staff and thus represent their recollection and interpretation of protocol adherence. While this project did include direct observations of protocol adherence, that component was started later in 2020 and very few observations occurred before the explosion. The specific staff sampled each week depended on a number of factors including availability to give an interview and the kind of service they were leading (with the intent of sampling from a range of service types). Because staff were not randomly chosen for interviews, there is likely some unmeasured noise in who completed interviews on any given day and their perceptions of protocol adherence. However, we do not have any reason to suspect that unmeasured variables correlated with service locations, and so should not affect the relative adherence between the different sites. But we have no way of determining if the explosion itself might have affected how staff in Beirut recalled protocol adherence during their services. Future research on nested crises testing the collective resilience vs. fragile rationalism orientations should attempt to utilize objective measures of their outcomes, either using direct observations or video recordings, to control for effects a crisis has on how people remember and report a given outcome.

There are several recommendations emerging from our research. First, while catastrophic disasters are often deeply disruptive, this analysis supports a policy of staying the course in terms of maintaining delivery of services and the basic health protocols around those services. Regardless of the disruption caused by the explosion, the NGO service providers still seemed to understand the importance of continuity in delivering medical, health, education and other refugee services—and there was no indication that those services were less well-utilized by the refugees themselves. The maintenance of COVID-19 prevention behaviors by both service providers and recipients indicates the interest in delivering and receiving services. We, therefore, recommend that donors continue funding agencies for continuity of deliverance of services to refugees. The NGOs participating in this study are embedded in communities and are personally known by service recipients, and our data indicated that the recipients and NGOs maintained relationships of trust despite the evident mistrust between the Lebanese government and the people in Lebanon. During times of crisis, this trust might be more important than peak functioning physical infrastructure.

Second, we noted that our data collectors actually increased data collection after the disaster. Indeed, the data collection and the meetings with our international research team seemed to provide a needed maintenance of routine and purpose in the midst of the post-explosion chaos. This leads to the recommendation that research organizations involved in evaluation and study of humanitarian assistance follow the lead of those on the ground. It may well be the case, in the context of the cascading crises like in Lebanon, that local researchers benefit from continued work even in the wake of a catastrophic event. The continuation of routine and the reason for contact with those outside the local context may well be welcome. Third, in the context of multiple-cascading disasters, donors and humanitarian agencies should not assume that practices on the ground will change dramatically in the wake of a major disaster that makes international media headlines. For the Lebanese people, the explosion was disruptive and caused loss of life and humanitarian infrastructure, but it was only the most recent in a long experience of crises. The resilience of the Lebanese people is inherent in understanding the need to carry on with day-to-day tasks amidst apparent catastrophe.

Lastly, we recommend that humanitarian actors normalize evidence-based health practices within service provision such that these practices become habit, especially during times of nested crises. Those norms can become protective habits against the disruptive effects of future shock events, such that even in the face of additional disasters, service providers and recipients will continue to follow safety protocols.

## Data Availability Statement

The raw data supporting the conclusions of this article will be made available by the authors, without undue reservation.

## Ethics Statement

The studies involving human participants were reviewed and approved by Institutional Review Board, Michigan State University. Written informed consent for participation was not required for this study in accordance with the national legislation and the institutional requirements.

## Author Contributions

SN, EK, and SG contributed to research design and implementation, data analysis and interpretation, and writing manuscript. RM and AG contributed to research design and implementation, interpretation of results, and writing manuscript. SM-P contributed to data analysis and writing manuscript. All authors contributed to the article and approved the submitted version.

## Funding

Funding for this project was provided by Elrha Learning and Research for Humanitarian Assistance and Michigan State University (grant #51551).

## Conflict of Interest

The authors declare that the research was conducted in the absence of any commercial or financial relationships that could be construed as a potential conflict of interest.

## Publisher's Note

All claims expressed in this article are solely those of the authors and do not necessarily represent those of their affiliated organizations, or those of the publisher, the editors and the reviewers. Any product that may be evaluated in this article, or claim that may be made by its manufacturer, is not guaranteed or endorsed by the publisher.

## Footnotes

^1^Amnesty International. “*The Worlds' Refugees in Numbers”*. Available online at: https://www.amnesty.org/en/what-we-do/refugees-asylum-seekers-and-migrants/global-refugee-crisis-statistics-and-facts/

^2^While Turkey's lira has been steadily depreciating vis a vis the US dollar since 2018, it was not until late 2021 that the current devaluation crisis happened, which was well after we completed data collection.

^3^MSU was the IRB of record, with permission for this given by the participating NGOs.
